# Comparison of clinical characteristics between COVID-19 and H7N9 fatal cases: An observational study

**DOI:** 10.3389/fpubh.2022.1047362

**Published:** 2022-11-24

**Authors:** Hui Jiang, Fangchao Liu, Ta-Chien Chan, Jinfeng Yin, Ruowen Huang, Li Shen, Shengjin Tu, Liang Kang, Wen Liu, Na Zhao, Di Zhang, Wangli Xu, Weimin Li, Shelan Liu, Chaolin Huang

**Affiliations:** ^1^Beijing Chest Hospital, Capital Medical University, Beijing, China; ^2^Beijing Tuberculosis and Thoracic Tumor Research Institute, Beijing, China; ^3^Research Center for Humanities and Social Sciences, Academia Sinica, Taipei, Taiwan; ^4^Beijing Normal University School of Mathematical Sciences, Beijing, China; ^5^Wuhan Jinyintan Hospital, Tongji Medical College, Huazhong University of Science and Technology, Wuhan, China; ^6^School of Ecology and Environment, Anhui Normal University, Wuhu, China; ^7^Center for Applied Statistics, School of Statistics, Renmin University of China, Beijing, China; ^8^Department of Infectious Diseases, Zhejiang Provincial Centre for Disease Control and Prevention, Hangzhou, Zhejiang, China

**Keywords:** COVID-19, H7N9, fatal cases, clinical course, protective factors, survival time

## Abstract

**Objective:**

The outbreak of COVID-19 in 2020 is reminiscent of the H7N9 outbreak in 2013, which poses a huge threat to human health. We aim to compare clinical features and survival factors in fatal cases of COVID-19 and H7N9.

**Methods:**

Data on confirmed COVID-19 and H7N9 fatal cases identified in mainland China were analyzed to compare demographic characteristics and clinical severity. Survival curves were estimated by the Kaplan–Meier method and compared using log-rank tests and a restricted mean survival time model. A Cox regression model was used to identify survival factors in fatal cases of COVID-19 and H7N9.

**Results:**

Similar demographic characteristics were observed in fatal cases of COVID-19 and H7N9. The proportion of fatal cases of H7N9 receiving antibiotics, antiviral drugs, and oxygen treatment was higher than that of COVID-19. The potential protective factors for fatal COVID-19 cases were receiving antibiotics (HR: 0.37, 95% CI: 0.22–0.61), oxygen treatment (HR: 0.66, 95% CI: 0.44–0.99), and corticosteroids (HR: 0.46, 95% CI: 0.35–0.62). In contrast, antiviral drugs (HR: 0.21, 95% CI: 0.08–0.56) and corticosteroids (HR: 0.45, 95% CI: 0.29–0.69) were the protective factors for H7N9 fatal cases.

**Conclusion:**

The proportion of males, those having one or more underlying medical condition, and older age was high in COVID-19 and H7N9 fatal cases. Offering antibiotics, oxygen treatment, and corticosteroids to COVID-19 cases extended the survival time. Continued global surveillance remains an essential component of pandemic preparedness.

## Introduction

The emergence in 2013 of novel avian influenza A H7N9 virus posed a pandemic threat to humans at the time, when human cases of infection from the virus occurred during annual winter–spring epidemics in mainland China (1). Fortunately, in September 2017, the successful development of an H5/H7 bivalent inactivated vaccine for chickens eliminated human infection with H7N9 virus (2, 3), and only three H7N9 cases have been reported since 1 October 2017 (2). Nevertheless, between 2013 and 30 September 2017, 1398 H7N9 cases and 560 H7N9 fatal cases were reported through the national surveillance system for notifiable infectious diseases in mainland China.

In December 2019, a novel coronavirus disease 2019 (COVID-19) outbreak occurred in Wuhan, China (4). Subsequently, outbreaks of human infections with severe acute respiratory syndrome-coronavirus-2 (SARS-CoV-2) occurred in 220 countries around the world (5). On 31 January 2020, the World Health Organization declared the COVID-19 outbreak to be a public health emergency of international concern (6). As of 13 September 2021, more than 21 million cases of COVID-19 had been reported globally, including 4,443,898 fatal cases around the world (7).

Here, we summarize the survival time and causes of clinical course changes in fatal cases of COVID-19 in mainland China compared with confirmed cases of H7N9 virus infections in the same region.

## Methods

### Study design and participants

This retrospective study includes a total of 290 fatal cases with COVID-19 at Jinyin-tan Hospital between 29 December 2019 and 24 April 2020. All cases were diagnosed based on the diagnosis and treatment protocol of the National Health Commission of the People's Republic of China (7th edition). Of these 290 fatal cases, 239 tested positive for SARS-CoV-2 by RT-PCR, while the remaining 51 were clinically diagnosed with COVID-19. The criterion of clinically diagnosed COVID-19 was confirmed by epidemiological history and clinical manifestations.(8) Epidemiological history: (1) History of travel to or residence in Wuhan and its surrounding areas, or in other communities where cases have been reported within 14 days prior to the onset of the disease; (2) In contact with novel coronavirus infected people (with positive results for the nucleic acid test) within 14 days prior to the onset of the disease; (3) In contact with patients who have fever or respiratory symptoms from Wuhan and its surrounding area, or from communities where confirmed cases have been reported within 14 days before the onset of the disease; (4) Clustered cases (2 or more cases with fever and/or respiratory symptoms in a small area such families, offices, schools etc. within 2 weeks). Clinical manifestations: (1) Fever and/or respiratory symptoms; (2) The imaging characteristics; (3) Normal or decreased white blood cell count, normal or decreased lymphocyte count in the early stage of onset. Clinically diagnosed COVID-19 case has any of the epidemiological history plus any two clinical manifestations or all three clinical manifestations if there is no clear epidemiological history.

In addition, we collected individual records of all 114 laboratory-confirmed H7N9 fatal cases in Zhejiang province from 18 March 2013 to 30 September 2017 from an integrated electronic database managed by Zhejiang CDC. Zhejiang province had the largest number of H7N9 cases. China required every identified H7N9 case to be reported to China CDC within 24 h via a national surveillance system for notifiable infectious diseases. Diagnostic confirmation of H7N9 infection was done either by the isolation of H7N9 virus or a positive real-time reverse-transcription polymerase chain reaction (RT-PCR) assay for H7N9 virus in a respiratory specimen (9).

#### Data collection

We collected the epidemiological, clinical, laboratory, and clinical management data for 290 fatal COVID-19 cases and 114 fatal H7N9 cases from Jinyin-tan Hospital's information system and Zhejiang province's integrated electronic database, respectively, using standardized forms. We also collected illness onset, diagnosis, and hospital admission times.

#### Statistical analysis

Descriptive statistical methods were adopted to analyze the continuous variables and categorical variables for H7N9 and COVID-19 cases, respectively. The differences between the two groups were compared by c^2^ test. We used the Kaplan–Meier (KM) method to estimate the survival rate and plot the survival curve. Before conducting a log-rank test between the different survival curves, we constructed a test for the proportional hazards (PH) assumption on groups by creating a time-dependent covariate in a PH model. A restricted mean survival time (RMST) model was adopted to plot the mean survival time curves. We were also able to use this model to compare the mean survival time in different groups relative to maximum survival time (τ). We used the Gaussian density estimation method, which is a nonparametric technique, to create a smoothing approximation of time-to-event distributions for illness onset to death and hospital admission to death. The Cox proportional hazards model was used to estimate the effect of covariates on survival time in H7N9 and COVID-19 cases.

Statistical analyses were performed using SAS 9.4 (SAS Institute, Cary, NC).

#### Ethical approval

The Ethics Review Committee of the Jinyin-tan hospital provided approval for this study (No: KY-2020-62·01). Additionally, patients' personal identifying information was anonymized to ensure privacy.

## Results

### Demographics and underlying medical conditions

A total of 290 fatal cases with COVID-19 and 114 fatal cases with H7N9 were analyzed in this study. Of those, 188 (64.83%) and 75 (65.80%) were male for COVID-19 and H7N9, respectively; the male-to-female ratios were 1.84:1 and 1.92:1, respectively. The median age was 68.00 years (IQR: 61.00–75.00) for COVID-19 cases and 65.00 years (IQR: 57.00–75.00) for H7N9 cases, and the age group distribution across the two groups was not significantly different (*p* = 0.125; Table 1 and Supplementary Figure 1A). In addition, there was no significant difference in gender and age in the different groups (Supplementary Figures 1B,C).

**Table 1:** Demographics and underlying medical conditions in fatal cases with COVID-19 and H7N9.

**Characteristics**	**COVID-19** **(*n =* 290, %)**	**H7N9** **(*n =* 114, %)**	**c^2^**	***p*** **-value**
Gender			0.033	0.855
Male	188 (64.83)	75 (65.80)		
Female	102 (35.17)	39 (34.20)		
Age (years, IQR)	68 (61–75)	65 (57–75)	0.085	0.770
Age group, years			7.213	0.125
< 55	43 (14.83)	20 (13.89)		
55–64	69 (23.79)	36 (25)		
65–74	91 (31.38)	29 (25.44)		
75–84	63 (21.72)	26 (22.81)		
≥85	24 (8.28)	3 (2.63)		
**Underlying medical conditions**				
Any	210/286 (73.43)	83/108 (76.85)	0.244	0.621
Hypertension	126/286 (44.06)	48/108 (44.44)	0.004	0.948
Diabetes	54/286 (18.88)	26/108 (24.07)	1.137	0.286
Cardiovascular disease	28/286 (9.79)	7/108 (6.48)	0.632	0.427
Tumor	20/286 (6.99)	6/108 (5.56)	0.064	0.800
Renal dysfunction	10/286 (3.50)	5/108 (4.63)	0.065	0.799
Chronic obstructive pulmonary disease	6/286 (2.10)	0/108 (0.00)	1.089	0.297
One chronic medical condition	116/286 (40.56)	45/108 (41.67)	0.035	0.852
Two chronic medical conditions	61/286 (21.33)	26/108 (24.07)	0.266	0.606
Three chronic medical conditions	37/286 (12.94)	7/108 (6.48)	2.547	0.111
Hypertension + diabetes	36/286 (12.59)	14/108 (12.96)	0.000	1.000
Hypertension + cardiovascular disease	19/286 (6.64)	5/108 (4.63)	0.230	0.632
Cardiovascular disease + diabetes	8/286 (2.80)	1/108 (0.93)	0.510	0.475

IQR, inter-quartile range.

There was no difference between the prevalence of underlying medical conditions for COVID-19 compared with H7N9 cases. Hypertension (44.06 vs.44.44%), diabetes (18.88 vs.24.07%), and cardiovascular disease (9.79 vs.6.48%) were the most common underlying medical conditions for both COVID-19 and H7N9. In addition, the proportion of fatal COVID-19 cases with three chronic medical conditions was higher than that of H7N9 fatal cases, although the *p*-value was not significant (Table 1).

### Times to events

**Figure 1:**
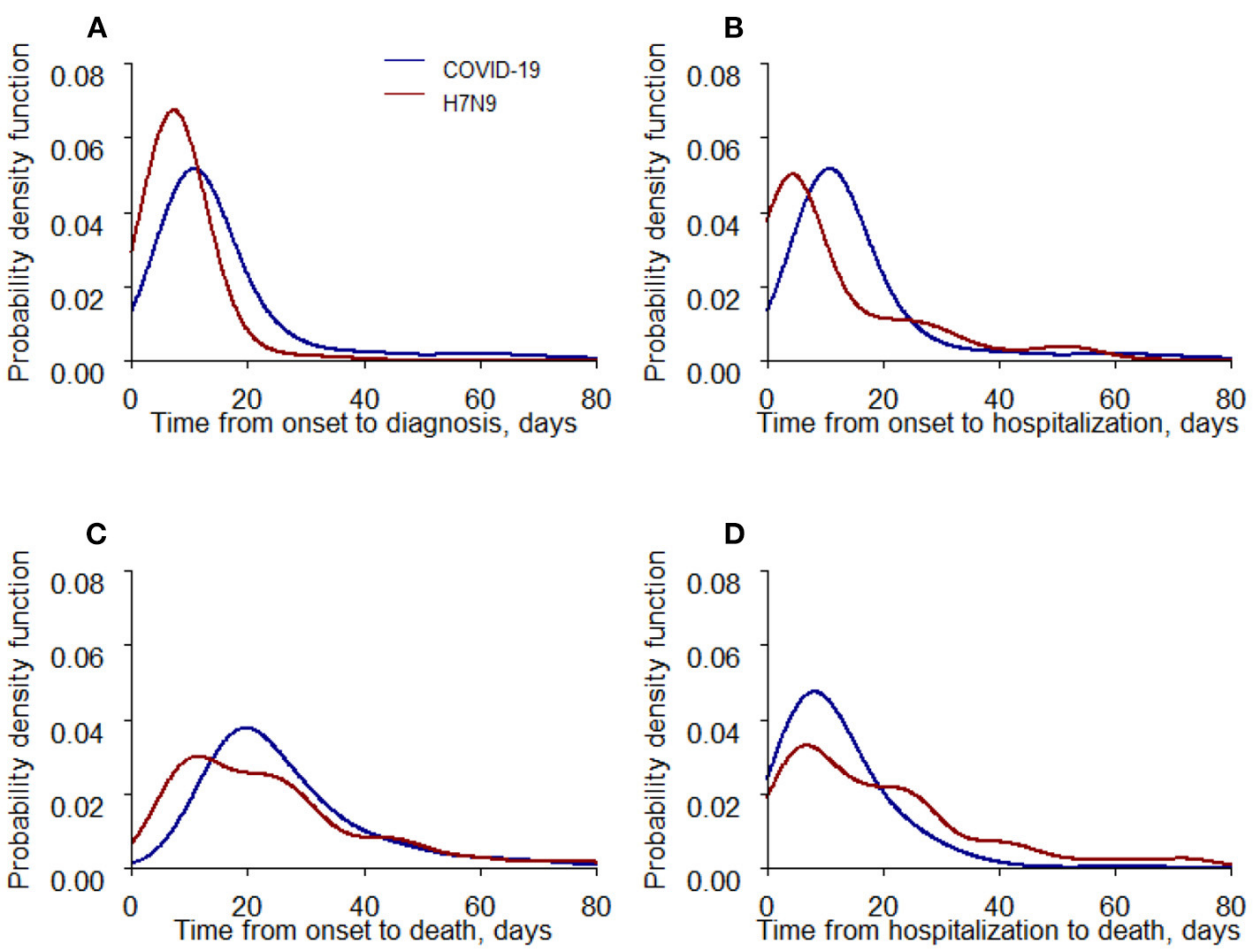
Time-to-event distributions of COVID-19 and H7N9 fatal cases. **(A)** Days from illness onset to diagnosis. **(B)** Days from illness onset to hospital admission. **(C)** Days from illness onset to death. **(D)** Days from hospital admission to death.

The median time from illness onset to diagnosis and from illness onset to hospital admission was different between the COVID-19 and H7N9 groups (*p* < 0.050); patients in the COVID-19 group had a longer time from illness onset to both diagnosis and hospital admission (11.00 and 11.00 days vs.7.00 and 6.00 days; Figures 1A,B). In addition, although the *p*-value was significantly different (*p* = 0.03686), the time from illness onset to death in the COVID-19 group was only 1 day longer than in the H7N9 group (23.00 days vs.22.00 days; Figure 1C), and the time from hospital admission to death was shorter in the COVID-19 group than that in the H7N9 group (10.00 days vs.15.00 days, *p* = 0.001; Figure 1D).

### Treatment and complications

From the perspective of treatment, although most COVID-19 and H7N9 fatal cases received antibiotics, antiviral treatment, and oxygen treatment, the proportion of H7N9 cases using the above treatments was significantly higher than that of COVID-19 cases, and the difference between the two groups was statistically significant (*p* < 0.05). However, the proportion of fatal COVID-19 cases (64.48%) was higher than that of H7N9 fatal cases (55.74%). In addition, the proportion of H7N9 fatal cases complicated with acute respiratory distress syndrome was significantly higher than that of fatal COVID-19 cases (25.23 vs.6.55%, *p* < 0.001) (Table 2).

**Table 2:** Treatment and complications in fatal cases with COVID-19 and H7N9.

**Characteristics**	**COVID-19** **(*n =* 290, %)**	**H7N9** **(*n =* 114, %)**	**c^2^**	***p*** **-value**
Received antibiotics	272 (93.79)	108/108 (100.00)	5.65	0.017
Received corticosteroid	187 (64.48)	34/61 (55.74)	1.30	0.254
Received antiviral drugs	161 (55.52)	87/105 (82.86)	23.51	< 0.001
Oseltamivir	35 (12.07)	75/87 (86.21)	174.44	< 0.001
Received ECMO	3 (1.03)	13/69 (18.84)	37.43	< 0.001
Received oxygen treatment	258 (88.97)	87/89 (97.75)	5.41	0.020
Received mechanical ventilation	201 (69.31)	71/81 (87.65)	9.97	0.002
**Complications**				
Respiratory failure	184 (63.45)	71/107 (66.36)	0.175	0.676
ARDS	19 (6.55)	27/107 (25.23)	24.84	< 0.001

ARDS, acute respiratory distress syndrome; ECMO, extracorporeal membrane oxygenation; IQR, inter-quartile range.

### Survival time from hospital admission and illness onset to death

**Figure 2:**
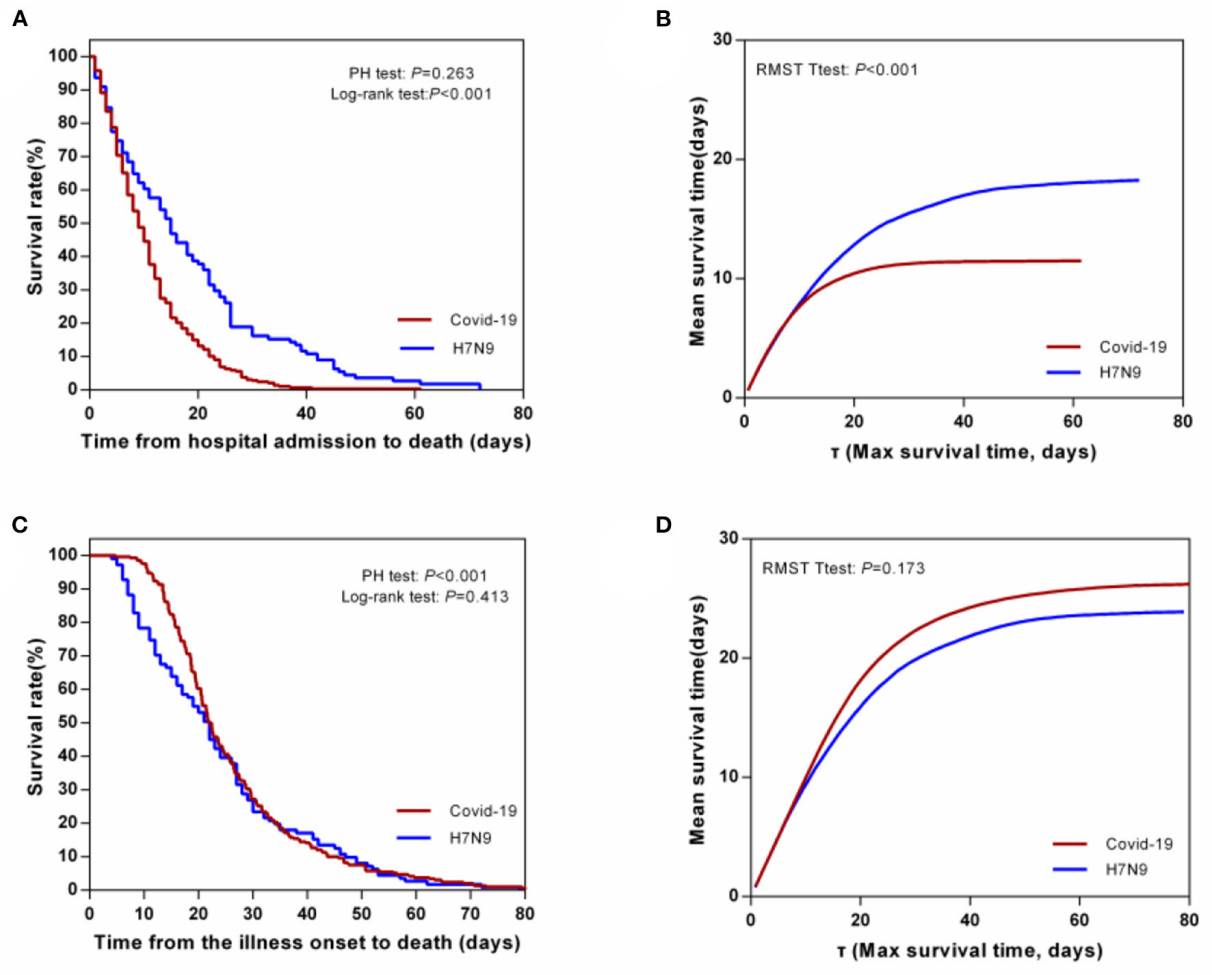
Survival time from hospital admission and illness onset to death by log-rank test and RMST curves. **(A)** Survival time from hospital admission to death by log-rank test. **(B)** Survival time from hospital admission to death by RMST curves. **(C)** Survival time from illness onset to death by log-rank test. **(D)** Survival time from illness onset to death by RMST curves.

Based on the time from hospital admission to death, a log-rank test showed that the survival time in hospital for H7N9 cases was longer than that for COVID-19 cases (*p* < 0.001; Figure 2A), and this difference was also observed in RMST curves (*p* < 0.001; Figure 2B). However, when using illness onset as the starting point for survival time, the difference between the two KM curves was not statistically significant (*p* = 0.413), and the mean survival time for COVID-19 cases was not statistically higher than that for H7N9 cases within maximum survival time (Figures 2C,D).

### Factors associated with survival time interval from hospital admission to death

The effect of covariates on the survival time for COVID-19 cases from hospital admission to death was assessed using a Cox proportional hazards model, and the potential protective factors included receiving corticosteroids, antibiotics, and oxygen treatment, after adjusting for other covariates. The risk of death among COVID-19 cases receiving antibiotics or oxygen treatment was significantly lower than among the cases who did not receive these treatments (HR: 0.37, 95% CI: 0.22–0.61 and HR: 0.66, 95% CI: 0.44–0.99, respectively). The risk of death among the COVID-19 cases who received corticosteroids was about half of that among those who did not receive corticosteroids (HR: 0.46, 95% CI: 0.35–0.62). The hazard of death in hospital among the H7N9 cases who receiving antivirus or corticosteroid was less than among the COVID-19 cases (HR: 0.21, 95% CI: 0.08–0.56 and HR: 0.45, 95% CI: 0.29–0.69, respectively). Receiving corticosteroid showed a protective effect both in the COVID-19 and the H7N9 cases. In addition, the time interval from hospital admission to death (median, IQR, days) was generally consistent with the HRs (Table 3).

**Table 3:** Forest plot of factors associated with survival time interval based on hazard ratio (HR) from hospital admission to death.

	**COVID-19**			**H7N9**		
**Parameter**	**Time interval from hospital admission to death (median, IQR, days)**	***p*** **-value**	**HR (95% CI)**	**Time interval from hospital admission to death (median, IQR, days)**	***p*** **-value**	**HR (95% CI)**
Age (per 10 years)	-	0.80	1.01 (0.92–1.12)	-	0.3524	1.09 (0.91, 1.3)
Time from onset to admission (days)	-	0.18	1.01 (1.00–1.02)	-	0.1568	1.04 (0.99, 1.09)
Male	10.40 (4.71–14.79)	Ref	Ref	14.0 (5.0, 25.0)	Ref	Ref
Female	9.46 (5.62–15.62)	0.56	0.93 (0.71–1.20)	16.0 (7.0, 30.0)	0.7311	0.93 (0.6, 1.44)
Not received antibiotics	3.66 (2.60–5.63)	Ref	Ref	6.0 (2.5, 11.5)	Ref	Ref
Received antibiotics	10.40 (5.69–15.65)	< 0.01	0.37 (0.22–0.61)	15.0 (6.0, 26.0)	0.3442	0.57 (0.18, 1.82)
Not received antivirus	9.63 (5.49–15.48)	Ref	Ref	1.0 (1.0, 2.0)	Ref	Ref
Received antivirus	10.42 (5.57–15.62)	0.56	1.16 (0.71–1.89)	15.0 (6.0, 26.0)	0.002	0.21 (0.08, 0.56)
Not received oxygen treatment	5.55 (2.66–12.58)	Ref	Ref	6.5 (3.0, 9.0)	Ref	Ref
Received oxygen treatment	10.41 (5.75–15.66)	0.04	0.66 (0.44–0.99)	16.0 (6.0, 26.0)	0.2583	0.66 (0.31, 1.37)
No underlying medical conditions	10.08 (5.61–20.46)	Ref	Ref	15.5 (7.5, 26.0)	Ref	Ref
Underlying medical conditions	9.47 (5.43–13.73)	0.15	1.23 (0.93–1.62)	14.0 (5.0, 26.0)	0.3719	0.8 1(0.51, 1.29)
Not received corticosteroid	7.39 (4.42–11.48)	Ref	Ref	6.0 (3.0, 18.0)	Ref	Ref
Received corticosteroid	12.53 (7.78–19.72)	< 0.01	0.46 (0.35–0.62)	21.0 (13.0, 30.0)	0.0003	0.45 (0.29, 0.69)

CI, confidence interval.

## Discussion

Human infection with H7N9 virus and SARS-CoV-2 were two respiratory infectious disease pandemics in China in recent decades that posed a major threat to public health, since these viruses may acquire mutations that enable efficient and sustained human-to-human transmission and lead to pandemic. Therefore, in this study, we compared the clinical processes of the two infectious diseases and confirmed their significantly different survival times by KM and RMST models. We found that male, having underling medical condition, older age were high-risk groups of COVOD-19 and H7N9 infection. Antibiotics, oxygen treatment, and corticosteroids were protective factors for fatal COVID-19 cases. In contrast, antiviral drugs and corticosteroids were the protective factors for H7N9 fatal cases.

We found that both COVID-19 and H7N9 fatal cases were mostly male, older, and with at least one underlying condition. Vaccine is the most effective method for preventing infectious disease, especially among high-risk populations. The H5/H7 bivalent inactivated vaccine for chickens was first used in September 2017, and the H7N9 virus isolation rate in poultry dropped by 93.3% following vaccination (4). Since most H7N9 cases had avian transmission, and human to human infection was limited, the avian vaccine was also effective in blocking human infections with H7N9 virus, and only three further cases of infection have been reported since September 2019. However, the current pandemic's SARS-CoV-2 pathogen spreads more widely and quickly and is highly transmissible from human to human (10), which leads to it posing a pandemic threat to human beings (11) and having a greater impact on the world. Therefore, effective vaccination of humans is particularly important as a means to prevent and block transmission. In China, as of 26 August 2021, more than two billion doses of COVID-19 vaccine had been distributed, and nearly 890 million people had completed the vaccination program. In the latest round of epidemics, the vaccination of COVID-19 has played a definite role in controlling it.

Early detection, diagnosis, and treatment form the basis for controlling and eliminating infectious diseases and improving survival opportunity. For H7N9 cases, early detection can control the disease process as much as possible and reduce the proportion of severity and death. However, for COVID-19 cases, early detection and diagnosis are not only to reduce severity and death but also to reduce the transmission of the SARS-CoV-2 virus. In the early stages of the COVID-19 epidemic, this new and emerging infectious disease was not fully understood, chaos, and resource limitations, so the time interval from illness onset to death was shorter, and that from illness onset to diagnosis was longer. However, greater understanding of COVID-19 and the continuous refinement and improvement of diagnostic criteria and improved sensitivity of detection kits not only accelerated the diagnosis of COVID-19 cases but also facilitated timely and accurate prevention and control. Nevertheless, false-negative test results may occur in up to 20–67% of cases (12); therefore, highly clinically suspicious patients should not rely solely on the results of RT-PCR tests, and clinical and epidemiological investigation should be carefully considered (13). In addition, the sensitivity of testing varies with the timing of testing relative to exposure (12). It is estimated that such sensitivity is 62% on the day of symptom onset and 80% on the third day after that, but it falls to only 33% 4 days after exposure (12, 14, 15). Therefore, timely sampling for detection is an important method for reducing false negative tests. Moreover, high-risk groups need to increase the number of their RT-PCR tests and to appropriately extend their isolation period.

At present, although there is no evidence to show that specific drug treatments are effective against suspected or confirmed cases with COVID-19 or H7N9, antiviral therapy and organ support therapy are the cornerstone of the treatment of severe cases with both diseases (16, 17). Our study indicated that more than half of the fatal COVID-19 and H7N9 cases received antibiotics (93.79 and 100.00%), antiviral drugs (55.52 and 82.86%), corticosteroids (64.48 and 55.74%), and oxygen treatment (88.97 and 97.75%) after hospital admission. Our study found that, without specific drug treatment for COVID-19 and H7N9, corticosteroid was a protective factor. This has been proven in research at Oxford University, where the latest study showed the ability of dexamethasone to reduce the risk of death by 54% in cases with COVID-19 requiring ventilation (18). In addition, recent research has indicated that the appropriate use of corticosteroids together with other remedies should be beneficial for severe cases of COVID-19 and H7N9 to prevent ARDS development (19, 20). Separately, severe COVID-19 cases are susceptible to secondary bacterial infection; therefore, a combination of antibiotics can also prolong the survival time of fatal cases and decrease the risk of death of COVID-19 cases requiring oxygen inhalation by 20% (18). For H7N9 cases, early initiation of antiviral therapy in patients is an important method to delay severity and death (17).

Surveillance is the most effective early warning method for emerging infectious diseases, and effective public health emergency management can reduce the adverse impact of emerging infectious diseases (21). In response to the 2003 severe acute respiratory syndrome outbreak, China established the National Notifiable Infectious Disease Surveillance System (NNIDSS) for 39 infectious diseases (22). The establishment of NNIDSS strengthened the construction of China's system for infectious diseases and public health emergencies (23) and played an important role in the outbreak of H7N9 avian influenza and the COVID-19 epidemic (21). However, infectious diseases have emerged one after another in recent years, and the existing surveillance system does not seem to be sufficient for the early warning of emerging infectious diseases. The Chinese government needs to consider further how to improve the existing surveillance system and its early warning role.

This study has some limitations. First, we only collected data on fatal cases, and mild and moderate cases were not included in the study; thus, a comprehensive comparison of survived and deceased cases was not possible. Second, we only collected information on early fatal COVID-19 cases, limiting our ability to characterize the differences between different variants. Third, some potential factors associated with survival time were not collected, which may cause bias. However, the most important factors have been collected.

In conclusion, we have described the clinical characteristics of fatal cases with COVID-19 and H7N9. We found the proportion of males, those having one or more underlying medical condition, and older age were high in these fatal cases. Moreover, from the perspective of individual treatment, offering antibiotics, oxygen treatment, and corticosteroids to COVID-19 cases extended the survival time. However, antiviral therapy and corticosteroids were more effective in H7N9 cases. Last, continued global surveillance of COVID-19 and human infections with avian influenza A viruses remains an essential component of pandemic preparedness.

## Data availability statement

The raw data supporting the conclusions of this article will be made available by the authors, without undue reservation.

## Ethics statement

The studies involving human participants were reviewed and approved by the Ethics Review Committee of the Jinyin-tan hospital. Written informed consent for participation was not required for this study in accordance with the national legislation and the institutional requirements.

## Author contributions

SL and HJ conceived, designed, and supervised the study. CH, LS, ST, LK, WL, NZ, and DZ collected and cleaned the data. FL, HJ, RH, and JY analyzed the data. HJ and FL wrote the drafts of the manuscript. SL, HJ, WL, and T-CC interpreted the findings. SL and CH commented on and revised the drafts of the manuscript. All authors have read and approved the final manuscript.

## Funding

This work was supported by Zhejiang Scientific and Technological Major Project under the 2020 Emergency Grant (Grant Number 2020C03124) entitled Key Technologies for Prevention and Control of Pneumonia Caused by Novel Coronavirus Infection, the Zhejiang University Special Scientific Research Fund for COVID-19 Prevention and Control (Grant Number 2020XGZX047), and the Fundamental Research Funds for the Central Public-Interest Scientific Institution (Grant Number 2022-PT320-01).

## Conflict of interest

The authors declare that the research was conducted in the absence of any commercial or financial relationships that could be construed as a potential conflict of interest.

## Publisher's note

All claims expressed in this article are solely those of the authors and do not necessarily represent those of their affiliated organizations, or those of the publisher, the editors and the reviewers. Any product that may be evaluated in this article, or claim that may be made by its manufacturer, is not guaranteed or endorsed by the publisher.

## Supplementary material

Supplementary Figure 1 Age distribution of COVID-19 and H7N9 fatal cases. **(A)** Age distribution of COVID-19 and H7N9 fatal cases by gender. **(B)** Age distribution of all COVID-19 fatal cases by gender. **(C)** Age distribution of all H7N9 fatal cases by gender.
