# Supplementary figures and images for: Comparison of clinical characteristics between COVID-19 and H7N9 fatal cases: An observational study
Source: Front Public Health. 2022 Nov 24;10:1047362. doi: 10.3389/fpubh.2022.1047362 (PMC9729836; doi:10.3389/fpubh.2022.1047362)

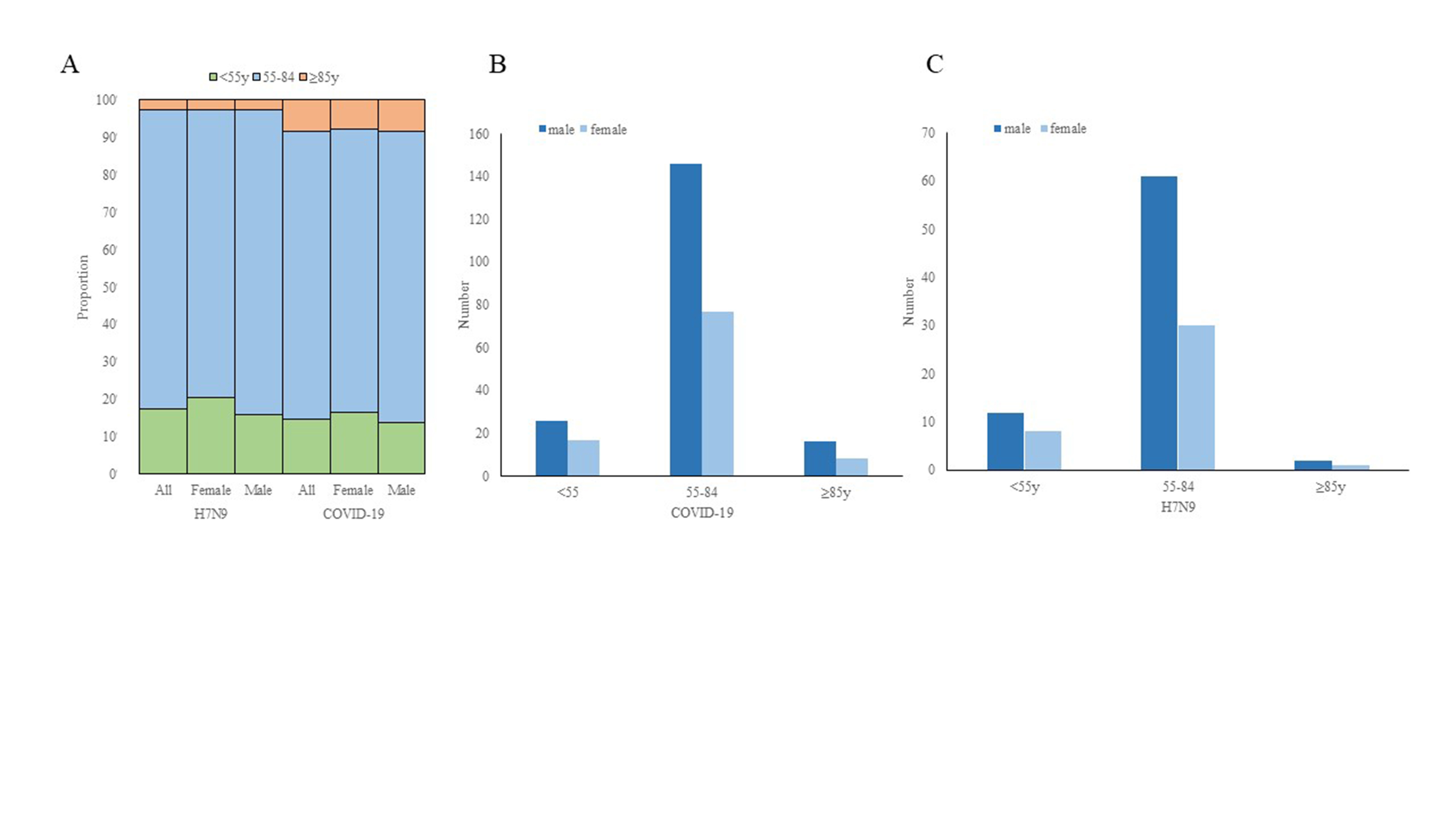

Supplement: Supplementary Figure 1 — Age distribution of COVID-19 and H7N9 fatal cases. (A) Age distribution of COVID-19 and H7N9 fatal cases by gender. (B) Age distribution of all COVID-19 fatal cases by gender. (C) Age distribution of all H7N9 fatal cases by gender. [file Image_1.JPEG]
